# Long Term Changes of the Axis of the Lower Limb After Chiari Pelvic Osteotomy—A Retrospective Analysis of 111 Osteotomies After 34 Years

**DOI:** 10.3390/jcm14041039

**Published:** 2025-02-07

**Authors:** Eleonora Schneider, Katharina Metzinger, Markus Schreiner, Jennifer Straub, Kevin Staats, Christoph Böhler, Reinhard Windhager, Catharina Chiari

**Affiliations:** 1 Department of Orthopedics and Trauma Surgery, Division of Orthopedics, Medical University of Vienna, Waehringer Guertel 18-20, 1090 Vienna, Austria; katharina.metzinger@gmail.com (K.M.); markus.schreiner@meduniwien.ac.at (M.S.); jennifer.straub@meduniwien.ac.at (J.S.); kevin.staats@meduniwien.ac.at (K.S.); christoph.boehler@meduniwien.ac.at (C.B.); reinhard.windhager@meduniwien.ac.at (R.W.); catharina.chiari@meduniwien.ac.at (C.C.); 2 Orthopedic Hospital Speising, Speisinger Straße 109, 1130 Vienna, Austria

**Keywords:** Chiari pelvic osteotomy, leg length difference, alignment, mechanical axis, long term changes, total hip arthroplasty, total knee arthroplasty, preoperative planning

## Abstract

**Background/Objectives**: The Chiari pelvic osteotomy (CPO) creates a bony roof by medialization of the acetabulum, thus improving the biomechanics of dysplastic hip joints. Long-term results have already been examined in various studies. However, the impact on the axis of the lower limb has not been investigated yet. The aim of this study was the analysis of changes in the alignment of the lower limb and leg length caused by a CPO and, consecutively, the impact on conversion total hip arthroplasty and primary knee arthroplasty. **Methods**: A total of 85 patients with 111 CPOs were clinically examined, patient reported outcome measures collected, and long leg standing radiographs analysed according to Paley. **Results**: The patients were examined an average of 34 years (±7.8; 23–53) after CPO. Unilaterally operated patients (N = 59 hips) showed a pathological MAD in 71% (N = 42) on the operated side (90% valgus, 10% varus). On the unaffected side, we could identify 56% (N = 33 hips) pathological cases (70% valgus, 30% varus). When patients underwent CPO bilaterally (N = 52 hips), the MAD was abnormal in 34 operated hips (65%; 97% valgus, 3% varus). If a leg length discrepancy occurred after the operation, the pelvis and the femur contributed the most to the total leg length discrepancy. **Conclusions**: Shifts in the mechanical axis following a CPO must be considered in order to inform patients appropriately preoperatively and quantified postoperatively by performing long leg standing radiographs in order to plan following joint replacement therapy adequately and maximize the chance of a successful long-term outcome on a functional level as well as for the patient’s satisfaction.

## 1. Introduction

Changes in the alignment of the lower limb in patients diagnosed with developmental dysplasia of the hip (DDH) are well documented [1,2,3]. Controversy remains on the long-term effects on angular deformities of the knee and ankle following proximal femoral varus osteotomies (PFVOs) or periacetabular osteotomies (PAOs) [4,5]. Also, valgus deformity of the knee after total hip arthroplasty in patients with severe dysplasia remains a concern [6]. While many studies have analysed the short and long-term results of the CPO, to the best of our knowledge, none of these have investigated the impact of the CPO on the alignment of the lower limb [7,8,9,10,11,12,13,14].

The Chiari pelvic osteotomy (CPO) was first performed by Karl Chiari at the Vienna University Clinic of Orthopaedics in 1952. The surgical principle of the CPO is a medial displacement of the intact hip joint, thus creating a better roof for coverage of the femoral head. The joint capsule is interposed between the bony roof and the femoral head and forms fibrocartilage over time. The technique was developed because of unsatisfying results of the previously popular shelf procedures [15], which were able to build a roof above the femoral head but not to medialize its position, which is crucial to achieve normal biomechanical function by restoring the physiologic lever arm of the pelvitrochanteric muscles [16]. The correct technique is to perform the osteotomy in an oblique fashion, ascending about 10° from lateral to medial. Thus, the centre of the hip joint is not only shifted medially but also cranially, which has an impact on the anatomical and mechanical axis of the lower limb [16,17]. Nowadays, reorienting osteotomies of the acetabulum like the periacetabular osteotomy (PAO) described by Ganz [18] or the Toennis triple osteotomy [19] are the preferred methods for the correction of dysplastic hip joints. The CPO still has a role as a salvage procedure, mostly used for the reconstruction of incongruent or severely dysplastic hip joints with very short and steep sockets.

As patients who were previously undergoing CPO are increasingly in need of joint replacement therapy (i.e., total hip arthroplasty (THA) and/or total knee arthroplasty (TKA)), the aim of our study was, therefore, to examine the effects on the alignment and leg length of the lower extremity on the long run in order to support surgeons in precise planning and conducting joint replacement therapy.

## 2. Materials and Methods

### 2.1. Patient Selection

The study was conducted in accordance with ethical standards, and approval for the study was gained by the institutional review board (EK Nr 1035/2012). All patients gave written consent prior to participation. We screened patient charts and radiographs from all patients who had undergone a CPO at our department between 1953 and 1986 and invited them to attend a follow-up examination. The cut-off chosen was 1986, as mostly Chiari himself or one of his direct trainees performed the CPOs until then, and, therefore, a surgeon-based bias could be minimized [17]. Patients with underlying neuromuscular disease or flexion contracture of the hip joint were excluded. Standardized, adequate long leg standing radiographs were prerequisite for inclusion. A total of 85 patients with 111 preserved CPOs (26 bilateral) were identified and clinically and radiologically analysed by the same examiner.

### 2.2. Study Population

The follow-up examination was conducted 34 years (±7.8; 23–53) after the CPO on average. Demographic details are listed in Table 1.

Some 48% of all CPOs were carried out after growth arrest (>16 years, n = 53), whereas 52% were younger than 16 years (n = 58). The indication for the CPO was developmental dysplasia of the hip in 77% (DDH; n = 85), subluxated or highly dislocated hips in 10% (n = 11), dysplastic osteoarthritis of the hip in 7% (n = 8), and dislocation of the hip as an infant in 6% (n = 7), respectively.

### 2.3. Clinical Evaluation

All patients were asked in detail about their medical history, focusing on the musculoskeletal system, and were invited to rate their overall satisfaction with their hip from 1 to 5 (1 = very good, 2 = good, 3 = satisfactory, 4 = sufficient and 5 = unsatisfactory). Physical examination included measurement of body height and weight, exact documentation of the gait (limping due to leg length difference, muscular insufficiency, pain or impaired range of motion (ROM)), Trendelenburg’s and Duchenne’s test, and the ROM (flexion, extension, abduction, adduction, inward rotation, and outward rotation). Pain levels were assessed by using the numeric rating scale (NRS [20]); the Harris Hip Score (HHS [21]) was used to assess the outcome of the hip surgery, and health status was measured by using the Short Form (SF) 36 questionnaire [22].

### 2.4. Radiological Evaluation

The bilateral long leg standing radiographs were the focus of analysis. Standing long-leg radiographs were obtained in the “stand-at-attention” position. Three images were consecutively acquired using a digital sensor, and stitching was undertaken using Philips Digital Diagnostics Software (Version 4.3, Philips Healthcare, North Ryde, Australia). The tube-to-knee distance was approximately 250 cm. Kilovoltage (kV) settings varied between 70 and 85 kV per cassette. Radiographs were performed using a Philips Optimus 80 generator (Philips Healthcare, North Ryde, Australia). The radiographs were collected from our institutional picture archiving and communication system (PACS, Sectra Imtec AB, Linköping, Sweden). The limb alignment was analysed according to Paley [23,24,25]. The abbreviations and standard values are listed in Table 2.

The grade of osteoarthritis was assessed by using the classification of Tonnis et al. (0 = no signs of osteoarthritis, 1 = mild, 2 = moderate, 3 = severe) [26].

**Figure 1:**
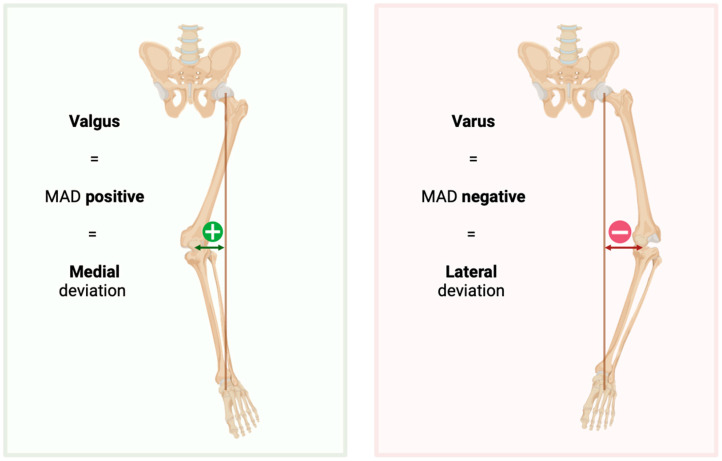
Description of Mechanical Axis Deviation calculation; MAD: mechanical axis deviation. Created in BioRender. Straub, J. (2025) https://BioRender.com/v59c708 (accessed on 6 January 2025).

Radiographs were measured digitally using TraumaCad^®^ software (Version 2.0, Brainlab^TM^) [27]. Each long leg standing radiograph was manually calibrated. The limb alignment analysis was measured bilaterally by marking six standardized landmarks per leg. The program then computed five angles (mLPFA, mLDFA, mMPTA, mLDTA, JLCA) and the mean axis deviation (MAD; Figure 1).

**Figure 2:**
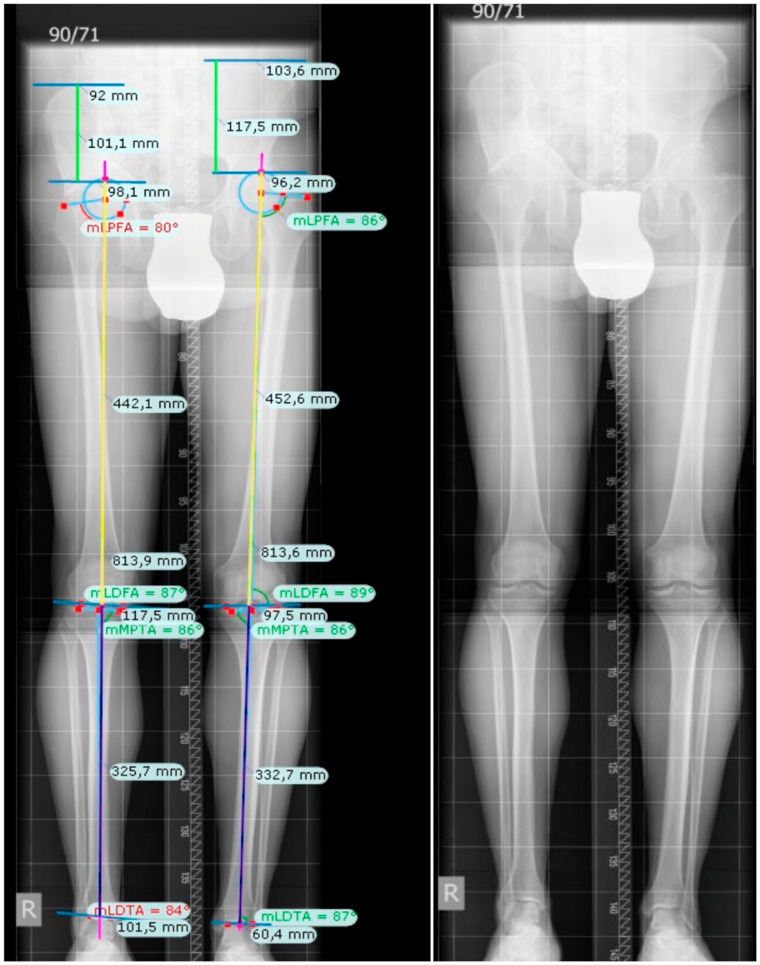
Exemplary radiograph with (**left**)/without (**right**) measurements for leg length difference and main angles; mLPFA mechanical lateral proximal femoral angle; mLDFA: mechanical lateral distal femoral angle; mMPTA: mechanical medial proximal tibial angle; mLDTA: mechanical lateral distal tibial angle.

Measurements along the mechanical axis were used to obtain the effective leg length discrepancy instead of the length differences of the bones. The malalignment-test according to Paley and Tetsworth was used to find the origin of the aberrance [28]. The femur can be identified as source of the deviation when the mLDFA shows aberrant results, such as <85° (lateral deviation of the MAD; i.e., valgus) or mLDFA > 90° (medial deviation of the MAD; i.e., varus). Abnormal MPTA values point to the tibia as derivation of the axial deviation (MPTA < 85° medial deviation of the MAD; i.e., varus; MPTA > 90° lateral deviation of the MAD; i.e., valgus). Abnormal JLCA (>3°) refers to ligaments and/or loss of cartilage as the origin. The total leg length difference (LLD) including the foot, the foot height difference, the total LLD including pelvis and foot, as well as the contribution of the pelvis to the LLD, were computed by measuring the parameters d1, d2, d3, and d4 manually with the ruler tool and inserting the parameters in Paley’s formula (Table 2) [29]. An example for all measurements is given in Figure 2. Furthermore, the differences of the right and left femur (=length right femur − length left femur) as well as the tibia (=length right tibia − length left tibia) and the difference of the sum of the right femur and tibia together (=(length right femur + tibia) − (length left femur + tibia)) were calculated. The standard (physiological) values for women and men, as reported in the literature, served as a controls, respectively [25,29,30].

### 2.5. Statistical Analysis

Descriptive statistics, including absolute and relative frequencies of nominal variables like sex, operated side, patients’ satisfaction, body mass index (BMI), abnormal gait, or the presence of Trendelenburg and Duchenne sign, were calculated and reported for all patients. Metric data were reported as means, standard deviation, and range. Further, analysis was carried out separately for unilaterally and bilaterally operated patients. Differences between continuous variables were assessed using *t*-tests, and Mann–Whitney–Wilcoxon tests were used in case of non-normal distributions. Differences between categorical variables were assessed using Chi Square tests, or Fisher’s exact test in case of less than 5 observations in any subgroup. All tests were applied in their two-sided version and conducted at a significance level of 0.05 using R version 4.2.2 (R Foundation for Statistical Computing; R Core Team. “R: A language and environment for statistical computing”). Foundation for Statistical Computing, Vienna, Austria (2013).

## 3. Results

### 3.1. Clinical Results

A total of 95% of all patients were satisfied with the CPO (70% very good, 22% good, 3% satisfactory). A total of 4% rated the outcome of the CPO as sufficient and 1% unsatisfactory. Overall, the gait was unimpaired in 52%, the Trendelenburg sign was positive in 31%, and the Duchenne sign was positive in 19%, respectively. In the subgroup of unilaterally operated patients, the gait was rated as normal in 46% (N = 27); limping due to LLD could be detected in 24% (N = 14), to muscular insufficiency in 22% (N = 13), and to pain one (2%) case. In 6%, the cause for limping was multifactorial. The average range of motion was 85° (±22; 10–130) flexion, 36° (±13; 0–60) abduction, 28° (±13; 0–60) adduction, 22° (±15; 0–80) outward rotation, and 15° (±12; 0–60) inward rotation. The NRS was 3 (±2.5; 0–8.7). The HHS showed 83 (±16; 34–100.86) points, the subscale HHS pain showed 35 (±10; 10–44), and the subscale HHS function showed 40 (±8; 14–47) points. Results of the SF-36 are summarized in Table 3.

### 3.2. Radiographic Results

**Figure 3:**
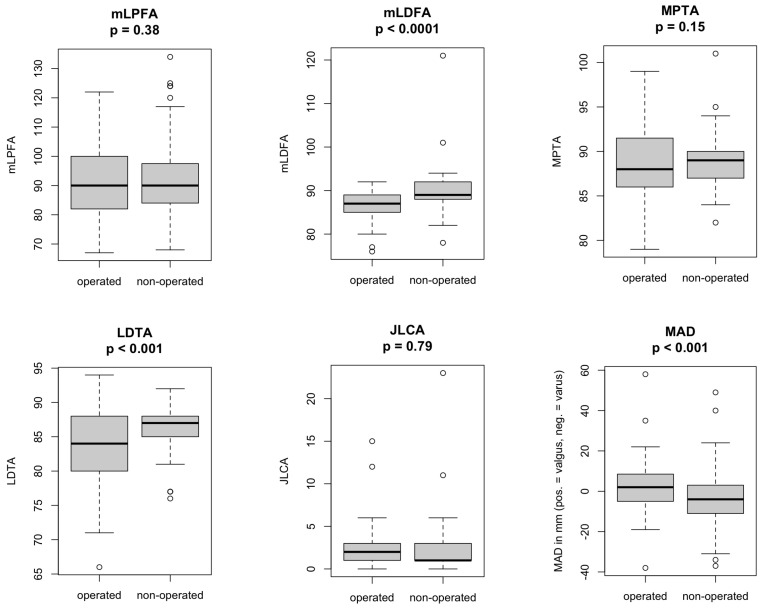
Overview of Limb Alignment Analysis Comparison of radiographic results in unilaterally operated patients applying the paired Wilcoxon signed rank test; mLPFA = mechanical lateral proximal femoral angle, mLDFA = mechanical lateral distal femoral angle, MPTA = medial proximal tibial angle, LDTA = lateral distal tibial angle, JLCA = joint line convergence angle, MAD = mechanical axis deviation.

Radiographic results are summarized in Figure 3. At the follow-up examination, the Toennis osteoarthritis grade was 2.4 (±0.7; 1–3) for the operated hip.

Analysing unilaterally operated patients only (N = 59), significant differences for the MAD (*p* = 0.0196), the mLDFA (*p* < 0.0001), and the total LLD (*p* = 0.0007) could be found. The mean MAD on the CPO side was 2.78 ± 13.99 (2; −38–58, range 96) and on the non-operated side was −3.66 ± 14.73 (−4; −37–49, range 86). Mean mLDFA on the CPO side was 86.3° ± 3.15° (87°, 76°–92°, range 16°) and on the non-operated side was 89.8° ± 5.38° (89°; 78°–121°; 43°), respectively. There was no significant difference in the MAD concerning the sex. On the operated side, the mean total LLD (including pelvis and foot) was 193 mm ± 58 mm (189 mm, 82–329 mm). Values for the non-operated side were 184 mm ± 57 mm (180 mm; 64–318). Within the cohort of unilaterally operated patients (N = 59), 34 patients (58%) had a clinically significant total LLD of more than 10 mm. The pelvis and the femur contributed most to the total LLD. The age at CPO had no significant influence on the development of LLD, but a trend towards more LLD could be detected for patients with dysplastic osteoarthritis of the hip as initial diagnosis.

Malalignment could be detected in the operated limb (7% varus (N = 4), 64% valgus (N = 38), 29% normal (N = 17)) more frequently than on the unaffected side (17% varus (N = 10), 39% valgus (N = 23), 44% normal (N = 26)). Focusing on the CPO side, in the malalignment test, varus (N = 4) was caused by the femur in one patient, the loss of cartilage/ligaments in one patient, or a combination of either the femur and tibia or the tibia and the cartilage/ligaments in one patient each. The valgus (N = 38) derived from the tibia in seven patients; the cartilage/ligaments or the combination of tibia and cartilage/ligaments in five patients each; the femur and tibia or the combination of femur, tibia, and cartilage/ligaments in three patients each; and solely the femur in two patients. In 13 patients, the source could not be further specified with the malalignment test. Applying the malalignment test on the unaffected side, varus (N = 10) was caused by the femur in four patients; the femur and ligaments/cartilage or the cartilage/ligaments only in two patients each; and the combination of femur, tibia, and ligaments/cartilage in one patient. Once, the source could not be further specified. Valgus (N = 23) was caused by the femur or the tibia and cartilage/ligaments in five patients each; the combination of femur, tibia, and cartilage/ligaments in three patients; the tibia in two patients; and the femur together with either the cartilage/ligaments or the tibia in one patient each. In six cases, the malalignment test did not reveal a specific origin of deviation.

## 4. Discussion

Plenty of literature exists on morphological changes in the knee joint associated with developmental dysplasia of the hip and the consecutive tendency for valgus axis deviation of the lower limb due to an abnormally shaped femoral condyle and a short lever arm of the proximal femur [3,31,32]. Sato et al., for example, investigated the differences in lower limb alignment and prevalence of knee osteoarthritis (OA) among patients with primary arthritis of the hip versus those with secondary arthritis of the hip due to DDH [3]. They found no differences in the prevalence of knee OA on the contralateral side, whereas the DDH patients showed significantly less knee OA and medial knee OA on the ipsilateral side. Then again, lateral knee OA tended to be higher in DDH on the ipsilateral side, but neither MAD nor LLD correlated with knee OA in those patients. Umeda et al. reported on less progression of knee OA on the side of hip OA due to a lateral shift in the mechanical axis [33]. In addition, several studies have been published recently on the post-operative changes after proximal femoral varus osteotomies and pelvic osteotomies as the PAO. Li et al. could show that a PAO combined with femoral osteotomy improved femoral offset, mechanical axis, and functional scores in patients with DDH, whereas excessive increase of femoral offset resulted in worse postoperative functional scores [4]. Liu et al. could not find any significant changes in DDH patients concerning the MAD, mLDFA, or LLD after more than 5 years of follow-up [5].

Others focused on the influence of THA on the axial and coronal alignment of the ipsilateral hip and knee [34,35,36,37]. In general, THA may lead to increased internal rotation of the hip, affecting gait, posture, and possibly the rate of dislocation.

Lucchini et al. reported that THA in low-grade dysplastic hips had no substantial impact on coronal or patellar alignment but neutralizing effects on femoral rotation, which could prove beneficial for dysplastic patients concerning anterior knee pain, stabilizing the joint and improving knee kinematics [34]. Akiyama et al. also investigated changes in the axial alignment of the ipsilateral hip and knee after THA. They hypothesized that, as underlying DDH leads to a lateral and superior shift of the femoral head as osteoarthritis progresses, changes in leg length as well as offset after THA may be greater in these patients [35]. They found both increased internal rotation associated with leg lengthening and lateral patellar tilt associated with younger age and leg lengthening after THA. A large variation in chronological changes could be observed, resulting in a decrease of approximately 2° over time within the follow-up of 2 years after index surgery [36]. Data on the impact on gait, rate of dislocation, or presence of anterior knee pain are missing. Pei et al. recently reported on the influence of THA on axial alignment of the lower limb in adults with unilateral DDH (Crowe IV) [38]. Patients presented significant valgus deformity preoperatively. Immediately after THA, the valgus was reduced or neutralized, and effects persisted over the FU period of two years.

Nonetheless, there is a paucity of data regarding the effects on limb alignment in the long term and, thereafter, on the influence of conversion THA or primary TKA, if necessary, after pelvic osteotomies. To the best of our knowledge, the impact of CPO on postoperative alignment and leg length discrepancies has not been the subject of investigation yet. Therefore, we report on changes of the mechanical axis and leg length differences of 111 osteotomies after a follow-up period of 34 years after CPO on average. In general, the procedure has been associated with good long-term results concerning pain, range of motion, and quality of life [12]. In our cohort, 95% of the patients were satisfied with the procedure, which is also reflected in good SF-36 results; the gait was normal in 52%, and the ROM was within the normal scope except for a reduced flexion of 85° on average. Pain was classified with 3 out of 10 at the NRS, and the Toennis grade of osteoarthritis was 2.4 on average 34 years after surgery.

Our data introduce several interesting findings. Firstly, impaired gait and a clinically significant LLD of more than one centimetre occurred more often in unilaterally operated patients. Due to its high prevalence in this cohort, as well as the impact on patients’ quality of life, good care must be taken of postoperative muscular insufficiency leading to a positive Trendelenburg sign. However, Windhager et al. showed that the Trendelenburg sign improved only in patients younger than 7 years at the time of CPO [11]. Moreover, LLD must be addressed and compensated in order to improve postoperative gait. Secondly, valgus deviation could be observed more frequently on the operated side but only mildly developed. It seems that medialization of the femoral head has a positive impact on the alignment of the affected side, as significantly less MAD could be observed on the operated limbs irrespective of uni- or bilaterally performed CPO. Therefore, it seems beneficial to address DDH at an early age in order to minimize negative effects on the alignment of the lower extremity. When comparing the length of femora on the operated versus the non-operated side, no significant difference could be detected. Thus, we assume, that the LLD is based on both a functional level resulting from the axis deviation combined with the cranialisation of the femoral head after the ascending osteotomy.

Our study has limitations. Firstly, the study was designed as a retrospective analysis, and the subgroups of the patients are relatively small. Secondly, as no pre-operative long leg standing films were available, we could not identify patients with axis deviation existing before the CPO or axis deviations for other reasons. In addition, analyses were based on 2-dimensional measurements (radiographs). Further 3-dimensional studies taking the triplanar realignment of the femur relative to the pelvis into consideration may be needed.

With this study, we provide important information about changes of the mechanical axis and the source of these changes, as well as possible leg length differences after CPO. Abnormal kinematics due to changes in muscular lever arms, leg length, and rotational centre might predispose patients to limping, instability, and the progress of osteoarthritis, requiring joint arthroplasty in the long run. We strongly suggest performing bilateral long leg standing radiographs prior to joint replacement therapy in all patients who undergo CPO. Chronological changes in the alignment of the lower extremity and ROM after CPO are frequent and must be taken into consideration for implant positioning. Restoring leg length and offset by total hip replacement might improve functional deficits acquired after CPO. As knee joint angles were altered significantly in the operated legs, a THA-first strategy and adaption of implant positioning of consecutive total knee replacements is recommended in patients who require both hip and knee arthroplasty.

## 5. Conclusions

Shifts in the mechanical axis as well as leg length discrepancy following a CPO must be considered and quantified in order to inform patients appropriately preoperatively and planned following knee or hip arthroplasties adequately in order to maximize the chance of a successful long-term outcome on a functional level, as well as for patients’ satisfaction.

## Figures and Tables

**Table 1:** Demographics.

Parameter	Value
Total number of patients	85
Total number of hips	111
Patients with unilateral CPO	59 (69%)
Sex (% female of all hips)	73 (86%)
Side (% right of all hips)	58 (52%)
BMI (kg/m^2^)	25 (±3.8; 18–39)
Age at follow-up (years)	52 (±9; 31–72)
Age at CPO (years)	18 (±11; 2–45)

Values given in mean (±standard deviation; range); BMI, body mass index; kg, kilograms; m^2^, square meter; CPO, Chiari pelvic osteotomy.

**Table 2:** Abbreviations and formula according to Paley and corresponding standard values.

Abbreviation	Nomenclature/Formula	Standard Values
mLPFA	Mechanical Lateral Proximal Femoral Angle	85–95°
mLDFA	Mechanical Lateral Distal Femoral Angle	85–90°
MPTA	Medial Proximal Tibial Angle	85–90°
LDTA	Lateral Distal Tibial Angle	86–92°
JLCA	Joint Line Convergence Angle	0–2°
MAD	Mechanical Axis Deviation	8 ± 7 mm medial
LLD (including foot)	(d_2_ − d_1_) + lift	d_1_ distance from top of X-ray film to femoral head right d_2_ distance from top of X-ray film to femoral head left
Foot height difference	Total LLD − [(F_1_ − F_2_) + (T_1_ − T_2_)]	F_1_ femoral length right F_2_ femoral length left T_1_ tibial length right T_2_ tibial length left
Total LLD (including pelvis and foot)	(d_4_ − d_3_) + lift	d_3_ pelvic length from Crest of Ileum to posterior inferior spine right d_4_ pelvic length from Crest of Ileum to posterior inferior spine left
Contribution of pelvis to LLD	(d_2_ − d_1_) − (d_4_ − d_3_)	

LLD—leg length difference.

**Table 3:** SF-36 Results.

Subscales	Results
Physical Functioning	64 (±27; 0–100)
Physical Role Functioning	69 (±42; 0–100)
Bodily Pain	56 (±28; 0–100)
General Health Perception	68 (±20; 25–100)
Vitality	60 (±17; 10–90)
Social Functioning	84 (±21; 12.5–100)
Emotional Role Functioning	81 (±38; 0–100)
Mental Health	74 (±18; 32–100)

Values in mean (±standard deviation; range); SF: short-form.

## Data Availability

The raw data supporting the conclusions of this article will be made available by the authors on request.
